# Genetic and pharmacological inhibition of inflammasomes reduces the survival of *Mycobacterium tuberculosis* strains in macrophages

**DOI:** 10.1038/s41598-020-60560-y

**Published:** 2020-02-28

**Authors:** Sathyavani Subbarao, Julia Sanchez-Garrido, Nitya Krishnan, Avinash R. Shenoy, Brian D. Robertson

**Affiliations:** MRC Centre for Molecular Bacteriology and Infection, Department of Infectious Disease, Flowers Building, Imperial College London, London, SW7 2AZ UK

**Keywords:** Infection, Innate immunity

## Abstract

*Mycobacterium tuberculosis* infection causes high rates of morbidity and mortality. Host-directed therapy may enhance the immune response, reduce tissue damage and shorten treatment duration. The inflammasome is integral to innate immune responses but over-activation has been described in tuberculosis (TB) pathology and TB-immune reconstitution syndrome. Here we explore how clinical isolates differentially activate the inflammasome and how inflammasome inhibition can lead to enhanced bacterial clearance. Wild-type, *Nlrp3*^−/−^/*Aim2*^−/−^, *Casp1*/*11*^−/−^ and *Asc*^−/−^ murine bone-marrow derived macrophages (BMDMs) were infected with laboratory strain *M*. *tuberculosis* H37Rv or clinical isolates from various lineages. Inflammasome activation and bacterial numbers were measured, and pharmacological inhibition of NLRP3 was achieved using MCC950. Clinical isolates of *M*. *tuberculosis* differed in their ability to activate inflammasomes. Beijing isolates had contrasting effects on IL-1β and caspase-1 activation, but all clinical isolates induced lower IL-1β release than H37Rv. Our studies suggest the involvement of NLRP3, AIM2 and an additional unknown sensor in IL-1β maturation. Pharmacological blockade of NLRP3 with MCC950 reduced bacterial survival, and combined treatment with the antimycobacterial drug rifampicin enhanced the effect. Modulating the inflammasome is an attractive adjunct to current anti-mycobacterial therapy that warrants further investigation.

## Introduction

Although significant strides have been made in reducing the disease burden attributed to TB, it remains a considerable cause of mortality. Current figures estimate that 1.6 million people died from TB in 2017^1^. Progress has been impeded by the rise in drug resistance and the HIV-epidemic. However, it is the ability of the bacterium to modulate its metabolism within the host environment^2^, enter non-replicating persistence^3^ and form biofilms^4^ in order to facilitate its own survival that leads to treatment difficulties^2^. Treatment of *M*. *tuberculosis* necessitates a prolonged multi-drug regimen^5^. Anti-microbials target actively replicating bacteria, but the intracellular population is composed of a mixed phenotype, requiring extended therapy to eradicate those bacterial populations that transiently and stochastically leave the slowly replicating state to enter an actively replicating state^5^. However, the extended treatment is associated with non-compliance and selection of resistant mutations.

To identify alternative anti-mycobacterial therapies efforts have been directed at altering the host immune response through host-directed therapy (HDT), which is to be used as an adjunct to standard quadruple therapy. Deregulated host immune responses may be counter-productive to bacterial killing and lead to tissue destruction, such that half of TB-survivors have some degree of persisting lung damage following successful microbiological cure^6^. Thus, the host response may be manipulated in two ways; firstly by augmenting bacterial killing and secondly by rebalancing the inflammatory response^7^. HDT is attractive as it does not have a specific anti-bacterial target in the same way as antimicrobials and therefore the risk of resistance is minimal^8^. The use of steroids for TB treatment in the 1950s is an early example of HDT^9^. Current evidence points to the efficacy of steroids during TB-meningitis and pericarditis^10^, and TB-immune reconstitution syndrome (IRIS)^11^. However, many individuals still have poor outcomes despite steroid treatment^12^. Cases of steroid refractory TB-meningitis that have responded to TNF-α blockers^13^ suggest that additional modulation of the innate and adaptive responses is needed.

Inflammasomes are signaling complexes that activate caspase-1, which in turn processes pro-inflammatory cytokines pro-IL-1β and pro-IL-18. Bioactive IL-1β production is regulated at multiple levels, including transcriptional regulation by NF-κB and post-translational cleavage of pro-IL-1β by caspase-1^14^. Transcription of pro-IL-1β can be initiated through the interaction between microbial ligands and surface toll-like receptors (TLRs). NOD and leucine-rich repeat containing proteins (NLRs), AIM2-like receptors or the protein PYRIN can respond to microbial or danger signals and assemble into inflammasomes along with the adapter protein ASC. The recruitment of caspase-1 into these complexes triggers protease activity and processing of substrates such as pro-IL-1β and pro-IL-18^15^. *M*. *tuberculosis* infection can activate NLRP3 inflammasomes in macrophages^16–19^. More recently, activation of the DNA receptor AIM2 via a process that requires the mycobacterial ESX-1 secretion system has been reported^20–24^ and one study showed lineage-specific induction of inflammasome-mediated inflammation that impacts on bacterial survival^25^. However, the mechanisms of inflammasome activation by clinical strains of *M*. *tuberculosis* remain poorly studied. We previously demonstrated differential induction of IL-1β by a panel of mycobacterial clinical isolates^26^, suggesting a difference in inflammasome activation. In this study, we further characterise inflammasome responses using these isolates and a panel of wild-type and inflammasome-deficient macrophages i.e. *Nlrp3*^−/−^/*Aim2*^−/−^, *Casp1*/*11*^−/−^ and *Asc*^−/−^ BMDMs, and find differential activation of these inflammasomes by clinical *M*. *tuberculosis* isolates as compared to the laboratory strain H37Rv. Mycobacterial survival is also affected by loss of inflammasome signalling pointing to a potential adjunctive role for inflammasome-blockade with antimycobacterial agents such as rifampicin. Thus, modulating inflammasomes could be a HDT against *M*. *tuberculosis*.

## Results

### Differential activation of inflammasomes and IL-1β processing by clinical isolates of *M*. *tuberculosis*

**Figure 1:**
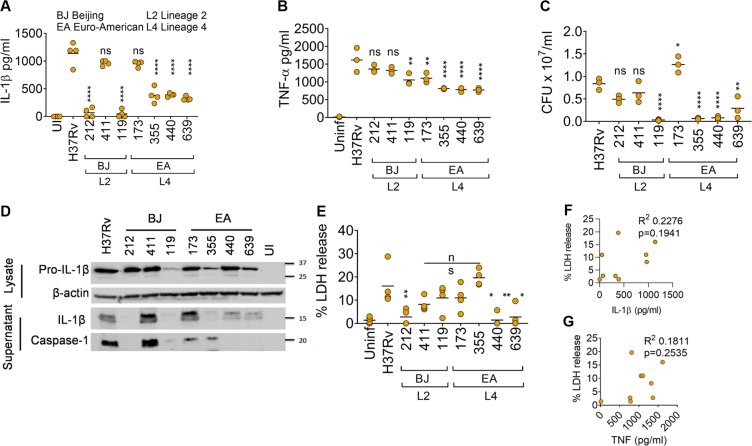
Differential release of IL-1β and TNF, activation of inflammasomes and macrophage cell death upon infection with clinical isolates of *M*. *tuberculosis*. (**A**,**B**) Quantification of IL-1β (**A**) and TNF (**B**) by ELISA from supernatants of immortalised C57BL/6 bone marrow-derived macrophages (iBMDMs) left uninfected (UI) or infected with H37Rv or indicated clinical *M*. *tuberculosis* isolates for 24 hours. (**C**) Colony forming units (CFU) of indicated *M*. *tuberculosis* strains from experiments in (**A,B**) measured at 24 h post-infection. (**D**) Representative immunoblots from iBMDMs infected with the indicated *M*. *tuberculosis* isolates for 24 hours. Images are representative of n = 3 experiments. (**E**) Cell death measured by the release of lactate dehydrogenase (LDH) from iBMDMs infected with indicated *M*. *tuberculosis* strains at 24 hours post-infection. (**F**,**G**) Plots showing the lack of correlation between cell death (LDH release) and ELISA for IL-1β (**F**) or TNF (**G**) from experiments in (**A**–**E**). Pearson’s correlation coefficient was calculated to test the linear dependence of IL-1β and LDH and TNF-α and LDH. *P ≤ *0*.*05*, **P ≤ *0*.*01*, ***P ≤ *0*.*001*, ****P ≤ *0*.*0001*, *ns* = *non-significant* by one-way ANOVA followed by Tukey’s multiple comparisons test for comparisons of clinical isolates with the H37Rv strain. Data and mean from n = 3–4 biologically independent experiments are shown in (**A**–**C**,**E**).

Previous studies have demonstrated distinct immunological changes following infection with clinical isolates and lineages of *M*. *tuberculosis*^25–29^. We infected immortalised wild-type murine bone marrow-derived macrophages (iBMDMs) using a panel of clinical isolates from the Euro-American and Beijing lineages as well as the H37Rv reference strain, followed by immunoblotting and ELISA for IL-1β and TNF at 24 h post-infection. All strains induced differential IL-1β release (Fig. 1A), with differences among lineages. These strains also induced differential levels of TNF, which is produced independently of inflammasomes; however, this did not mirror IL-1β release (Fig. 1B). For instance, even though the Beijing strains 212 and 119 elicited little IL-1β, they led to the production of comparable levels of TNF from macrophages (Fig. 1A,B). As the engagement of TLRs controls the expression of pro-IL-1β primarily through the MyD88 signalling pathway^30^, and these receptors may work cooperatively in phagocytosis^31^ we quantified bacterial survival in macrophages (Fig. 1C). There were marked difference in uptake of H37Rv versus other strains. Isolate 173 was taken up at higher levels and strains 119, 355, 440 and 839 were poorly phagocytosed. To assess pro-IL-1β and caspase-1 proteolysis due to the activation of inflammasomes, we performed immunoblot analyses. Immunoblotting revealed that in general, the Euro-American strains induced less caspase-1 and IL-1β maturation (Fig. 1D). The results from immunoblots for mature IL-1β were consistent with the results obtained by ELISA, which is less able to differentiate between pro and mature IL-1β in culture supernatants (Fig. 1A,D). Notably, marked differences were seen between H37Rv, 212 and 411 in their ability to induce caspase-1 activation and bioactive IL-1β release even though they were taken up by macrophages to similar levels (Fig. 1C,D). This pointed to differential activation of inflammasomes by these Beijing isolates (Fig. 1D). Inflammasomes are also responsible for pyroptotic cell death although inflammasome-independent cell death has been reported in *M*. *tuberculosis* infected macrophages^32,33^. We used lactate dehydrogenase (LDH)-release assays to measure cell death induced by *M*. *tuberculosis* in iBMDMs (Fig. 1E). Most *M*. *tuberculosis* strains induced cell death of macrophages however, cell death did not correlate with IL-1β release (Fig. 1F) (p = 0.1941) or TNF (Fig. 1G) (p = 0.2535). This indicated that caspase-1 activation and cytokine maturation are uncoupled from cell death during infection with clinical isolates of *M*. *tuberculosis*. We therefore decided to further characterise inflammasome activation by strains 212, 411 and H37Rv strains which survived in macrophages to the same degree and induced similar levels of pro-IL-1β and TNF.

### The adaptor protein ASC is essential for IL-1β production induced by *M*. *tuberculosis* infection

**Figure 2:**
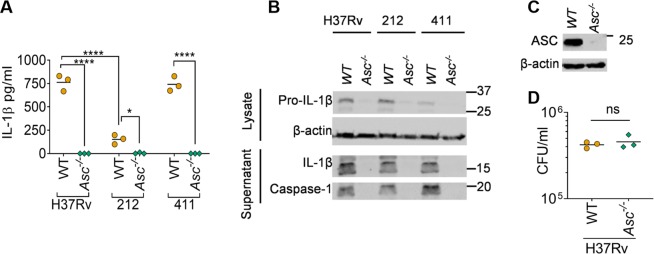
The adaptor protein ASC is necessary for caspase-1 activation in *M*. *tuberculosis-*infected macrophages. (**A**) ELISA quantification of IL-1β released from wildtype (WT) or *Asc*^−/−^ iBMDMs infected with the indicated *M*. *tuberculosis* isolates for 24 hours. (**B**) Representative immunoblots from iBMDMs infected with the indicated *M*. *tuberculosis* isolates for 24 hours. Images are representative of n = 3 experiments. (**C**) Representative immunoblots showing ASC and β-actin expression in WT and *Asc*^−/−^ iBMDMs. Images are representative of n = 3 experiments. (**D**) Uptake of *M*. *tuberculosis* H37Rv by WT and *Asc*^−/−^ macrophages at 4 h post-infection. *P ≤ *0*.*05*, ****P ≤ *0*.*0001*, *ns* = *non-significant* for indicated comparisons by two-way ANOVA followed by Tukey’s multiple comparisons test in **A** and two-tailed Student’s *t*-test in (**D**). Data and mean from n = 3 biologically independent experiments are shown in (**A**,**D**).

We infected immortalised wild-type and *Asc*^−/−^ BMDMs (iBMDMs) with the chosen clinical isolates and quantified IL-1β release by ELISA. IL-1β production was completely dependent on ASC (Fig. 2A). However, immunoblotting revealed that ASC deficiency also severely affected the abundance of pro-IL-1β upon *M*. *tuberculosis* infection (Fig. 2B,C). Uptake of H37Rv was comparable between the wild-type and ASC deficient cells (Fig. 2D), ruling out the role of phagocytosis in the reduced pro-IL-1β expression in *Asc*^−/−^ iBMDMs. However, previous work has indicated an *Nlrp3-* and *Casp1*-independent role for ASC in NF-κB activation in different settings^34^, indicating that a similar defect might be the reason for reduced pro-IL-1β expression in these cells upon *M*. *tuberculosis* infection; in contrast, other reports suggest that *Asc*^−/−^ iBMDMs do not have defects in NF-κB signalling^35–38^. Importantly, *Asc*^−/−^ cells showed no detectable caspase-1 activation after infection with H37Rv or strains 212 and 411 (Fig. 2B). Taken together, our experiments pointed towards the critical roles of ASC in inflammasome-independent pro-IL-1β expression and as an inflammasome adaptor during *M*. *tuberculosis* infection.

### Clinical *M*. *tuberculosis* isolates induce partial IL-1β processing in the absence of NLRP3 and AIM2

**Figure 3:**
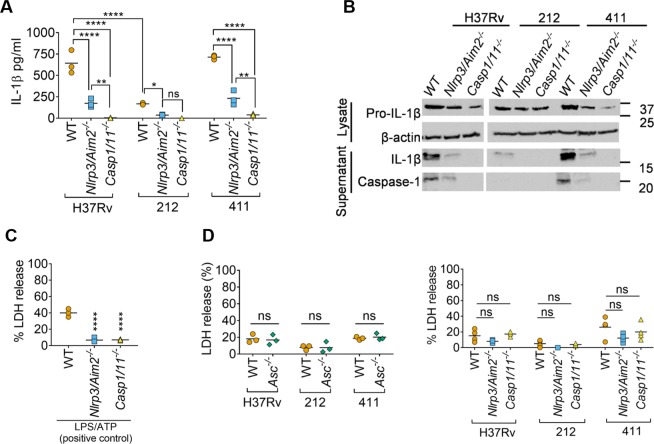
Clinical *M. tuberculosis* isolates can induce partial IL-1β processing in the absence of NLRP3 and AIM2 and inflammasome-independent cell death. (**A**) ELISA quantification of IL-1β released from wildtype (WT), *Nlrp3*/*Aim2*^−/−^ and *Casp1*/*11*^−/−^ iBMDMs infected with H37Rv or the indicated clinical *M*. *tuberculosis* isolates for 24 hours. (**B**) Representative immunoblots from iBMDMs infected with indicated *M*. *tuberculosis* isolates for 24 hours. Images are representative of n = 3 experiments. (**C**) Cell death measured by the release of lactate dehydrogenase (LDH) from iBMDMs. Cells were treated with LPS (3 h; 250 ng/ml) followed by ATP (5 mM; 60 min) as a positive control. (**D**) LDH release assay from macrophages infected with the indicated *M*. *tuberculosis* strains for 24 hours. *P ≤ *0*.*05*, **P ≤ *0*.*01*, ***P ≤ *0*.*001*, ****P ≤ *0*.*0001*, *ns* = *non-significant* for indicated comparisons in (**A,D**) by two-way ANOVA followed by Tukey’s multiple comparisons test, and one-way ANOVA for comparison with WT iBMDMs in **C**. Data and mean from n = 3 biologically independent experiments are shown in (**A**,**C**,**D**).

NLRP3 and AIM2 have been implicated in detecting *M*. *tuberculosis* infection in macrophages^16–24^. To assess their roles in detecting clinical *M*. *tuberculosis* isolates, we infected iBMDMs from *Nlrp3*^−/−^/*Aim2*^−/−^ and *Caspase1*/*11*^−/−^ knockout mice. Note that C57BL/6 *Casp1*^−/−^ cells also harbour a defective *Casp11* locus and are therefore labelled *Casp1*/*11*^−/−^ here. The double-deficiency of *Nlrp3* and *Aim2* led to a marked reduction in IL-1β release as measured by ELISA following *M*. *tuberculosis* infection, and a complete loss of IL-1β production was observed in *Casp1*/*11*^−/−^ cells (Fig. 3A). However, residual IL-1β release could be detected in *Nlrp3*^−/−^/*Aim2*^−/−^ cells infected with *M*. *tuberculosis* (Fig. 3A). In contrast to isolate 411 and H37Rv, which induced comparable IL-1β release in a partially *Nlrp3*/*Aim2*-dependent manner, isolate 212 elicited ~75% less IL-1β that was entirely *Nlrp3*/*Aim2-*dependent (Fig. 3A). Concordant results were obtained by immunoblotting for mature IL-1β and active caspase-1. Loss of *Nlrp3 and Aim2* severely reduced IL-1β maturation and caspase-1 activation, and IL-1β maturation was abolished in *Casp1*/*11*^−/−^ cells (Fig. 3B). Furthermore, the absence of *Asc*, *Nlrp3*/*Aim2* or *Casp1*/*11* had no impact on cell death upon infection with strains of *M*. *tuberculosis*, indicating that *M*. *tuberculosis*-induced cell death is inflammasome-independent (Fig. 3C,D). Taken together, our results show that *Casp1*/*11* and *Asc* are required for IL-1β processing following infection with *M*. *tuberculosis* clinical isolates. The residual caspase-1/11, ASC-dependent and NLRP3/AIM2-independent maturation of IL-1β in response to infection with H37Rv and isolate 411 suggests the involvement of an additional inflammasome sensor in detecting these bacteria.

### The NLRP3 inhibitor MCC950 and rifampicin cooperatively reduce IL-1β production by *M*. *tuberculosis* infected macrophages

**Figure 4:**
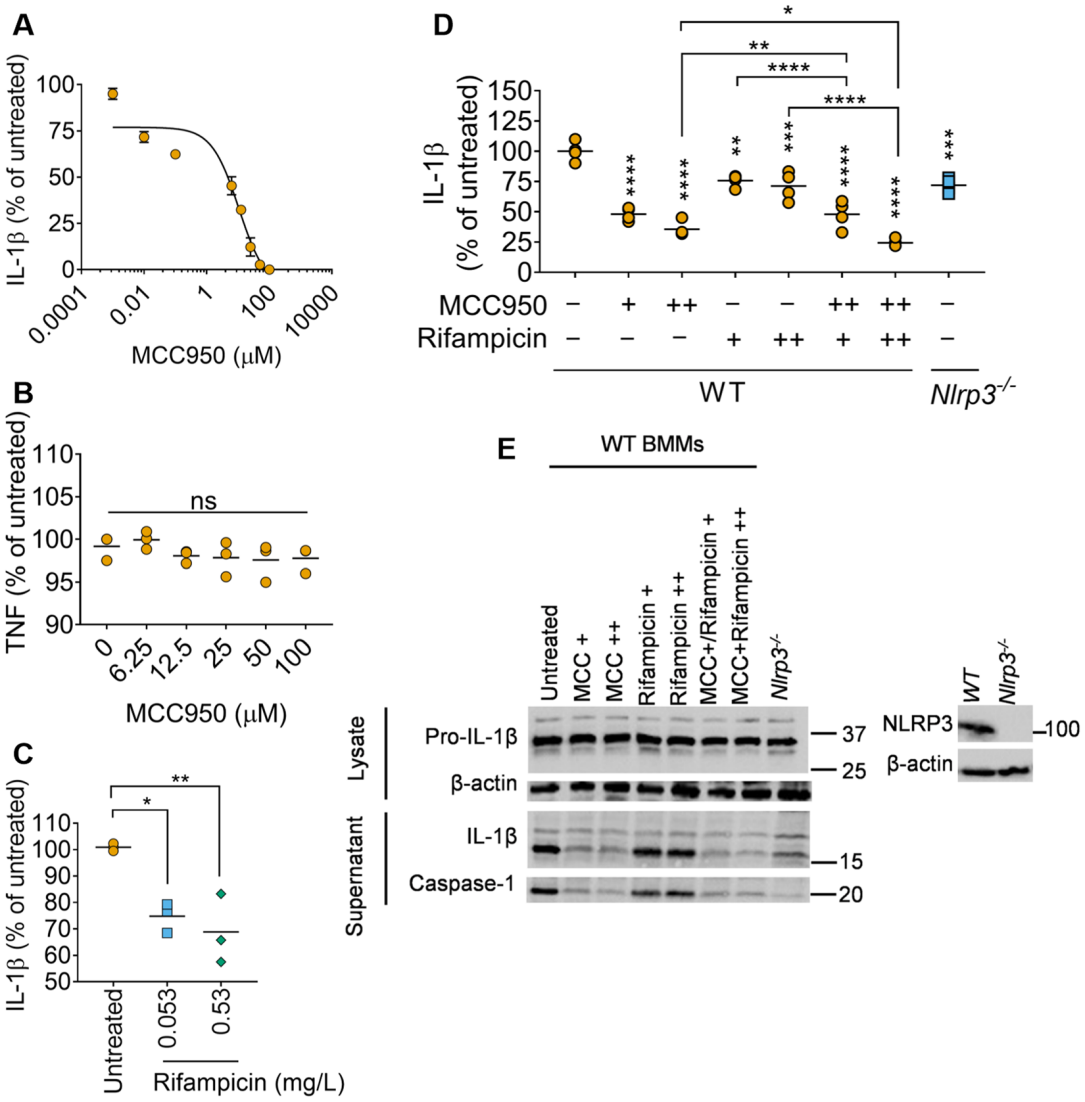
The NLRP3 inhibitor MCC950 and rifampicin cooperatively reduces IL-1β production by *M*. *tuberculosis* infected macrophages (**A-B**) Relative IL-1β (**A**) or TNF (**B**) released from wildtype iBMDMs infected with H37Rv for 24 h. iBMDMs were infected for 4 hours and then treated with NLRP3-inflammasome inhibitor MCC950 for an additional 20 h. (**C**) Relative IL-1β released from wildtype infected iBMDMs incubated in the presence of indicated concentrations of rifampicin. (**D**) ELISA quantification of IL-1β released from wildtype or *Nlrp3*^−/−^ iBMDMs infected with H37Rv in the absence or presence of MCC950 (+, 6.25 μM; ++, 12.5 μM) and/or rifampicin (+, 0.053 mg/L; ++, 0.53 mg/L). (**E**) Representative immunoblots from iBMDMs infected with *M*. *tuberculosis* H37Rv in the presence or absence of indicated drugs. Immunoblots on right show the lack of expression of NLRP3 in *Nlrp3*^−/−^ iBMDMs. Images are representative of n = 3 experiments. *P ≤ *0*.*05*, **P ≤ *0*.*01*, ***P ≤ *0*.*001*, ****P ≤ *0*.*0001*, *ns* = *non-significant* by one-way ANOVA followed by Tukey’s multiple comparisons test for comparisons with untreated samples. Mean ± sem are shown in **A**, and data and mean from n = 3 biologically independent experiments are shown in (**A**–**D**).

In order to explore the potential for a pharmacological approach to inflammasome manipulation, we tested MCC950, a small molecule inhibitor of the NLRP3 inflammasome^39^ that specifically inhibits activation of NLRP3 but not the AIM2, NLRC4 or NLRP1 inflammasomes, and has been used in several inflammatory settings *in vivo* and *in vitro*. For example, MCC950 inhibits the pro-inflammatory response following exposure to house dust mite-induced airway inflammation in mice^40^ and affords protection against influenza virus A *in vivo*^41^. MCC950 treatment of *M*. *tuberculosis* infected iBMDMs lead to a dose-dependent reduction in IL-1β release (Fig. 4A). The IC_50_ was ~5 µM and complete inhibition of IL-1β was achieved at 100 µM, at which concentration there may be off-target activity^39^. Importantly, no difference in TNF release was observed upon MC9550 treatment even at high concentrations (Fig. 4B), which mirrors previous findings^39^.

Adding adjunctive immune therapy to standard anti-microbial treatment is an attractive prospect that could reduce overall treatment duration, reduce tissue damage and improve patient outcome. Recent studies have shown that anti-inflammatory agents may satisfy these parameters^42,43^. We first identified the MIC of rifampicin using the resazurin microtitre (REMA) assay (0.026 mg/L), and treatment with both 2× and 20× the MIC led to similar reductions in IL-1β release (Fig. 4C), which verified that live, metabolically *M*. *tuberculosis* are responsible for eliciting IL-1β from iBMDMs. Wild-type iBMDMs were infected with H37Rv and treated with 6.25 µM or 12.5 µM MCC950, with or without 0.053 mg/L or 0.53 mg/L rifampicin for 24 h (Fig. 4D). As MCC950 specifically inhibits the NLRP3 inflammasome, we also infected *Nlrp3*^−/−^ iBMDMs as a control. Western blots confirmed the lack of NLRP3 in the *Nlrp3*^−/−^ iBMDMs (Fig. 4E). In agreement with previous results (Fig. 4A), treatment with 6.25 µM and 12.5 µM MCC950 led to 52 ± 5% and 64 ± 6% reduction in IL-1β release during *M*. *tuberculosis* H37Rv infection (Fig. 4D). Addition of rifampicin at 0.053 or 0.53 mg/L in combination with MCC950 further reduced IL-1β release compared to MCC950 alone (52 ± 6 or 76 ± 3% reduction, respectively; Fig. 4D). Immunoblots showed that reduced IL-1β release in the presence of MCC950 was due to reduced caspase-1 activation and IL-1β maturation (Fig. 4E). As expected, there was reduced but detectable IL-1β processing in *Nlrp3*^−/−^ iBMDMs in the ELISA and immunoblots (Fig. 4D,E). Taken together, combined treatment of *M*. *tuberculosis*-infected macrophages with rifampicin and the NLRP3-inhibitor MCC950 led to a greater reduction in IL-1β release as compared to treatments with MCC950 or rifampicin alone.

### MCC950 acts adjunctively with rifampicin and reduces *M*. *tuberculosis* survival

**Figure 5:**
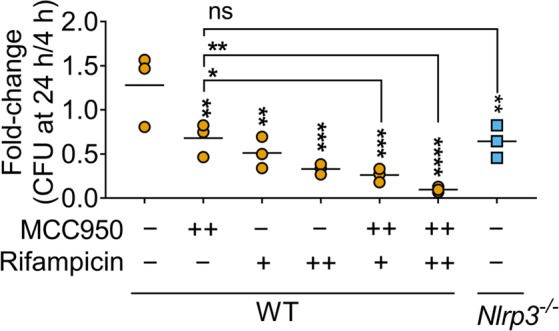
MCC950 alone and in combination with rifampicin reduces *M*. *tuberculosis* survival *in vitro*. Fold change in bacterial survival between 4 and 24 h post-infection in H37Rv infected iBMDMs is plotted. Cells were treated with MCC950 (++; 12.5 μM) and/or rifampicin (+, 0.053 mg/L; ++, 0.53 mg/L) as indicated. *P ≤ *0*.*05*, **P ≤ *0*.*01*, ***P ≤ *0*.*001*, ****P ≤ *0*.*0001*, *ns* = *non-significant* by one-way ANOVA followed by false-discovery-rate based correction for multiple comparisons for comparisons with untreated samples or indicated comparisons with MCC950-treated samples. Comparison between Rif + and Rif+/MCC950++ and between Rif ++ and Rif++/MCC950++ are not significant and omitted from the figure for clarity. Data and mean from n = 3 biologically independent experiments are shown.

The impact of MCC950 on NLRP3 in *M*. *tuberculosis*-infected macrophages led us to hypothesise that pharmacological inhibition would partially phenocopy the reduced *M*. *tuberculosis* survival observed in knockout cells. Indeed, bacterial survival was also reduced in *Nlrp3*^−/−^ cells pointing to an important role for this sensor in promoting *M*. *tuberculosis* survival (Fig. 5). MCC950 treatment reduced bacterial survival in wildtype macrophages to a similar extent as does genetic loss of *Nlrp3*, which ruled out off-target effects for MCC950 (Fig. 5). Importantly, like their effects on IL-1β production, treatment with rifampicin alone or in combination with MCC950 cooperatively reduced *M*. *tuberculosis* survival in wildtype macrophages (Figs. 4D and 5). To ensure that MCC950 does not directly inhibit mycobacterial growth, we performed the REMA assay using serially diluted concentrations starting at 100 µM MCC950 which did not reveal differences compared to untreated controls (data not shown). We therefore concluded that pharmacological inhibition of the NLRP3 inflammasome reduces *M*. *tuberculosis* H37Rv survival in macrophages.

### Effect of inflammasome inhibition on survival of *M*. *tuberculosis* clinical isolates

**Figure 6:**
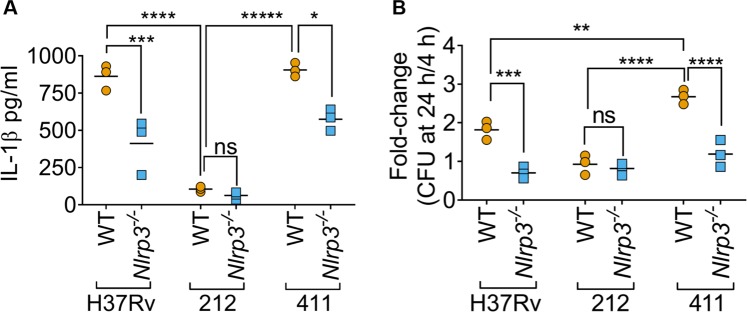
*M*. *tuberculosis* strain-dependent restriction of replication by inflammasomes. (**A**) ELISA quantification of IL-1β released from wildtype or *Nlrp3*^−/−^ iBMDMs infected with *M*. *tuberculosis* H37Rv and clinical isolates. **(B)** Fold-change survival of *M*. *tuberculosis* strains between 4 and 24 h post-infection in infected iBMDMs is plotted. *P ≤ *0*.*05*, **P ≤ *0*.*01*, ***P ≤ *0*.*001*, ****P ≤ *0*.*0001*, *ns* = *non-significant* by two-way ANOVA followed by Tukey’s test for indicated comparisons. Data and mean from n = 3 biologically independent experiments are shown.

We then asked whether MCC950 or loss of *Nlrp3* would affect bacterial survival and IL-1β induction by *M*. *tuberculosis* clinical isolates. As the NLRP3 inflammasome is druggable using MCC950 and loss of *Nlrp3* alone impacted H37Rv survival and IL-1β release, we decided to perform these experiments with *Nlrp3*^−/−^ rather than *Nlrp3*^−/−/^*Aim2*^−/−^ cells. We hypothesised that strains that elicit higher IL-1β, such as H37Rv and 411, do so in an *Nlrp3-*dependent manner and their survival would be more affected by loss of NLRP3 than that of isolate 212 which induces much less IL-1β release (Fig. 1). Indeed, H37Rv and isolate 411 released 52% and 37% less IL-1β upon infection of *Nlrp3*^−/−^ cells as compared to wildtype iBMDMs (Fig. 6A). However, IL-1β release induced by 212 infection was comparable in wildtype and *Nlrp3*^−/−^ iBMDMs (Fig. 6A). Like H37Rv which also activates inflammasomes, the survival of the inflammasome-activating isolate 411 was markedly reduced in *Nlrp3*^−/−^ cells at 24 h post-infection (Fig. 6B). Importantly, isolate 212, which does not trigger NLRP3-dependent IL-1β release, survived similarly in wildtype and *Nlrp3*^−/−^ iBMDMs (Fig. 6B). Taken together, pharmacological inhibition of NLRP3 with MCC950 led to a reduction in IL-1β release and bacterial survival which was also observed in *Nlrp3*^−/−^ iBMDMs.

## Discussion

TB was responsible for 1.6 million deaths in 2017^1^ thus efforts are being channelled into providing treatment regimens that are truly short-course and better tolerated. Host-directed therapies are now being considered as an adjunctive to antimicrobial drug treatment that may mitigate imbalances in the host immune response and additionally aid bacterial clearance^8^. Thus far, studies evaluating the utility of host-directed therapy in *M*. *tuberculosis* have focused on the lab-adapted strain H37Rv. In this study, we present data suggesting that inflammasome activation is strain-specific and this needs to be factored in when considering adjunctive therapy. Importantly, we also found that inflammasome inhibition may aid bacterial clearance following infection by *M*. *tuberculosis* strains that are detected well by the host inflammasome pathway.

Differences in cytokine induction have been shown for mycobacterial lineages^25,29^ and sub-lineages; in particular, significant differences within the Beijing-lineages^28,44,45^ have been demonstrated. In this study, we showed that clinical isolates induced variable IL-1β and TNF responses and that there are marked differences in bacterial uptake between different strains. The phenotypic differences seen in the clinical isolates may in part be attributed to variability in the mycobacterial cell envelope which should be investigated in the future. For example, examination of 17 mycobacterial strains showed an overexpression of *virS* in the Euro-American lineages which may indirectly play a role in modifying the mycolic acid components of the cell envelope and in turn affect bacterial uptake^46,47^.

Consistent with our previous study on differential IL-1β release by clinical *M*. *tuberculosis* isolates, here we showed that the production of the pro-IL-1β precursor and activation of caspase-1 both differ among strains. Notably, not all clinical isolates activated the inflammasome. A previous study proposed that *M*. *tuberculosis* inhibits AIM2 expression and thus inhibits that inflammasome^21^, which might underlie some of these differences. As with *M*. *tuberculosis*, strain-specific inflammasome activation has previously been observed during infection with various isolates of uropathogenic *Escherichia coli*^48^. It was demonstrated that inflammasome activation and IL-1β release by some *E*. *coli* strains was completely dependent on NLRP3 and ASC, with some of these strains activating the inflammasome to heighten virulence, which is associated with urinary tract infection pathology^48^. Indeed, Taxman *et al*.^34^ showed an essential role for ASC in the induction of IL-1β by TLR2, 4, 5 agonists, and live bacteria. ASC expression was sustained in *Porphyromonas gingivalis*-infected cells until cytokines were induced, and silencing ASC expression reduced NF-κB activation in response to *Porphyromonas gingivalis*-infection.

We also found residual caspase-1-dependent IL-1β maturation in cells lacking NLRP3 and AIM2, pointing towards the involvement of other inflammasome sensors. A recent study identified the NLRP7 inflammasome as a mediator of caspase-1 activation during *Mycobacterium bovis* infection^49^. Microbial lipopeptides signalling via TLR2 have also been shown to activate the NLRP7 inflammasome^50^. Although NLRP7 is a human protein, it is closely related to murine NLRP2, and knock outs of this sensor would shed light on the additional inflammasome involved in the response to *M*. *tuberculosis*. Indeed, a screen in human THP1 macrophages has identified additional NLRs involved *M*. *tuberculosis*-induced IL-1β production^51^.

We recently showed that caspase-1 and caspase-11 restrict the growth of cytosolic *Salmonella* in mouse macrophages^52^. Previous work has showed that overexpression of caspase-1 restricts *M*. *tuberculosis* replication^51^. A more recent study^25^ showed that *M*. *tuberculosis* from Lineage 4 show a higher rate of replication and induce more proinflammatory cytokines (IL-1β, IL-6, and TNF) in human macrophages than strains from lineages 1 and 5. An autocrine IL-1β-induced autophagy was observed, which surprisingly did not affect Lineage 4 mycobacterial survival. We found that MCC950 reduced IL-1β release and *M*. *tuberculosis* survival. Future studies using *Il1r1*^−/−^ cells or IL1R-agonists could verify whether autocrine/paracrine IL-1β-signalling plays a similar role in promoting autophagy in this system. Bacterial escape into the cytosol is detected by cGAS which stimulates the production of type I interferons through the adapter protein STING; cGAS also targets bacteria for autophagy^53^. A recent study showed that siRNA knockdown of AIM2 in J774A.1 cells increased LC3-II and reduced p62 levels following *Mycobacterium bovis* infection and inhibited bacterial replication *in vitro*^24^. This suggests that AIM2 potentially enhances bacterial survival by inhibiting autophagy. Furthermore, the authors showed increased levels of type I interferons due to activation of the STING pathway in AIM2-silenced cells and that exogenous type 1 IFN increased autophagy activation in wildtype macrophages. Whether *Nlrp3*^−/−^/*Aim2*^−/−^ iBMDMs exhibit enhanced autophagy should be verified in the future.

Previous studies have indicated that inhibition of NLRP3 signalling can downplay *M*. *tuberculosis* inflammatory reaction *in vitro*^54^,^55^. Similarly, following influenza A virus infection, NLRP3 appears to contribute to an early inflammatory environment which is protective, but this subsequently progresses to an excessive inflammatory response that is associated with increased mortality which can be mitigated by the use of MCC950^40,56^. Our results on the strain-specific impact of inflammasomes/MCC950 suggest that administration of inflammasome inhibitors should be undertaken if the infection is by a strain that elicits inflammasome activation. Future work should undertake a wider and systematic testing of clinical *M*. *tuberculosis* isolates for their ability to activate inflammasomes. It would be immensely useful to identify whether MCC950 has potential against such groups of strains as a treatment adjunct alongside antibiotics. In summary, we have shown that shown that clinical isolates of *M*. *tuberculosis* lead to a differential activation of the inflammasome and IL-1β processing, and that IL-1β can be produced in the absence of AIM2 and NLRP3. Further, for higher IL-1β-inducing strains, survival is affected by inflammasome activity. This highlights that host-directed therapy might have potential against a subset of *M*. *tuberculosis* strains and warrants future investigation with a larger set of strains.

## Methods

### Bacterial strains and culture conditions

All experiments with *M*. *tuberculosis* were carried out in a dedicated BSL3 facility in accordance with local rules approved at Imperial College London. The clinical isolates were cultured from frozen stocks that were a kind gift from Dr. Nitya Krishnan and Dr. Guy Thwaites^28^. Clinical isolates included representatives from the Beijing lineages (212, 411, 119) and Euro-American lineages (173, 639, 440, 355). The laboratory mycobacterial strain, H37Rv, was used as the reference strain. 500 μl bacteria were thawed into 10 ml liquid cultures and grown over 7 days in Middlebrook 7H9 media supplemented with 10% oleic acid-albumin-dextrose-catalase (OADC, BD), 0.05% Tween-80 (Sigma), 0.2% glycerol (VWR) at 37 °C in a shaking incubator.

### Cell culture

The cells used in this work were harvested from C57/BL6 mice; wild-type, *Nlrp3*^−/−^/*Aim2*^−/−^, provided by Dr. Katherine Fitzgerald^57^; *Nlrp3*^−/−^ and *Caspase1* /*11*^−/−^, provided by Dr. Richard Flavell^58^ and *Asc*^−/−^, provided by Dr. Vishwa Dixit^35^. BMDMs were immortalised as described before^59^ to generate iBMDMs which were used throughout the study. iBMDMs were grown in DMEM (Sigma) plus 10% heat-inactivated fetal calf serum (Labtech), 5 mM sodium pyruvate (Life technologies) and 10% L929 spent medium. The spent medium was obtained by growing L929 cells (a kind gift from Dr. Maximiliano Gutierrez, Francis Crick Institute) in DMEM to 100% confluence and removing the medium after 48 hours. BMDMs were seeded when 80% confluence was achieved, seeded in a 48-well tissue culture plate at 3.5 × 10^5^ cells/well and kept at 37 °C and 5% CO_2_ for all experiments.

### Macrophage infection

Macrophages were infected with log phase cultures of *M*. *tuberculosis* at a multiplicity of infection (MOI) of 5. After 4 hours, extra cellular bacteria was removed by washing macrophages with pre-warmed DMEM. To determine the intracellular bacterial load, macrophages were lysed at 4 and 24 hours post-infection, lysates serially diluted in PBS-0.05% Tween-80 and plated on Middlebrook 7H11 agar plates and colony forming units (CFU) was determined 3 weeks after incubation. Cells incubated with LPS (L7770, Sigma) for 3 hours followed by 5 mM ATP (Sigma) stimulation were used as a positive control for IL-1β and caspase-1 detection in the initial experiments. Additionally, after 24 hrs, cell supernatants were harvested and double-filtered through 0.22 μm filter (Corning Costar Spin-X centrifuge tube filters) at 10,000 × *g* for 5 minutes and frozen at −20 °C for ELISA experiments.

### Resazurin microtiter assay (REMA) assay for measuring the susceptibility of H37Rv to rifampicin and MCC950

The REMA assay identifies the minimum inhibitory concentrations of a drug^60^. Briefly, 100 μl of 7H9-broth was added to a sterile 96-well plate and serial two-fold dilutions of the drug were prepared within the broth (the highest concentrations for rifampicin and MCC950 were 1.96 mg l^−1^ and 100 mM respectively). Wells were inoculated with 100 μl of bacterial suspension such that the final OD was approximately 0.005. A growth control and sterile control was also included. The plate was covered with a lid and kept in a plastic box and kept at 37 °C incubator. After 7 days, 30 μl resazurin was added to the wells and the plate was re-incubated for 2 days. The change from blue resazurin to pink implied bacterial growth. The MIC was the lowest concentration that did not yield a change in colour^61^.

### Treatment with MCC950

MCC950 (previously known as CRID3^62^) was obtained from Tocris Biosciences, Cat. No. 5479. On the day of the experiment, BMDMs were infected with the bacterial strain and cells were washed after 4 hours with pre-warmed DMEM. Drugs were pre-diluted into their required working concentrations in Opti-MEM. Washed cells were then incubated in the presence of MCC950 for 24 hours.

### Cytokine detection by Enzyme-linked immunosorbent assay (ELISA)

Mouse TNF-a and IL-1β were quantified using commercially available ELISA kits for mTNF (e-Biosciences #88-7324) and mIL-1β (e-Biosciences #88-7013), following manufacturer’s instructions.

### Lactate dehydrogenase assay for cell cytotoxicity

Lactate dehydrogenase (LDH) was quantified using an LDH kit (Promega) according to the manufacturer’s instructions. LDH assays used untreated cells (0%) and 1% Triton-X100 (100%) to calculate percent LDH released.

### Antibodies used for immunoblotting

The following antibodies for immunoblotting were used. Anti-β-actin-HRP (sc-47778, Santa Cruz Biotechnology or A3854, Sigma-Aldrich), goat anti-mouse IL-1β (AF-401, R&D systems), anti-mouse caspase-1 (Casper 1, AG-20B-0042-C100, Adipogen), anti-ASC (AG-25B-0006, Adipogen) and anti-NLRP3 (AG-20B-0014, Adipogen). Secondary anti-mouse and anti-goat antibodies were obtained from Santa Cruz Biotechnology, anti-rabbit secondary antibody was from Thermo Fisher Scientific.

### Immunoblotting

For immunoblotting experiments, the supernatants from four wells per condition were filtered twice through 0.22 μm filters (Corning Costar Spin-X centrifuge tube filters) and pooled. The sample was desalted using spin desalting columns (87768, Thermo-scientific). A final volume of 800 μl was precipitated overnight with ice-cold acetone (ratio 1:4, v/v) and resuspended in 30 μl 2× Laemelli loading buffer (4 g SDS, 20% glycerol, 120 mM Tris pH 6.8, 0.01% bromophenol blue and 20 mM EDTA). Cell extracts were prepared by adding 100 μl Laemelli buffer with 1× protease inhibitor (Pierce), 1 mM PMSF and 5% β-mercaptoethanol to each well and duplicate samples were pooled. Prior to loading on an SDS-PAGE gel, both the lysate and supernatants were boiled at 100 °C for 10 minutes. 25 μl of each sample was loaded at on a 13.5% SDS-PAGE gel. Proteins were separated by SDS-PAGE using Tris-Glycine buffers and transferred onto PVDF membranes. Membranes were blocked for 2 hours at room temperature in 10% fat-free milk and incubated overnight at 4 °C with the appropriate antibodies. Secondary antibodies were added following washing of the membrane with PBS–Tween-80 (0.05%). The membrane was probed with antibodies against IL-1β and developed before being stripped and reprobed with antibodies against caspase-1. Finally, blots were stripped and re-probed with β-actin as a loading control.

### Statistical analysis

Statistical analysis was performed using GraphPad Prism 7. Matched parametric data was analysed using a two-tailed paired t-test, or One Way Analysis of Variance (ANOVA) for three or more groups followed by either Tukey’s post-hoc analysis or Dunnet’s multiple-comparison test (the choice of which depended on whether a known control was being compared against). Two-way ANOVA was used to determine the influence of two independent variables on a dependent variable and was followed by Tukey’s post-hoc analysis to ascertain this difference. Pearson’s correlation coefficient was used for assessing a correlation between IL-1β and LDH.
